# Blood pressure changes across pregnancy and postpartum: from physiological adaptations to pathological deviations

**DOI:** 10.3389/fcvm.2026.1953409

**Published:** 2026-10-28

**Authors:** Domenico Cozzo, Natacha Gassmann, Anne Dufey Teso, Belén Ponte, Thenral Socrates

**Affiliations:** 1 Service of Nephrology and Hypertension, Department of Medicine, University Hospitals of Geneva, Geneva, Switzerland; 2 Faculty of Medicine, University of Geneva, Geneva, Switzerland; 3 Service of General Internal Medicine, Department of Medicine, Geneva University Hospitals, Geneva, Switzerland; 4 Medical Outpatient Department and Hypertension Centre, ESH Hypertension Centre of Excellence, University Hospital Basel, Basel, Switzerland; 5 Department of Cardiology, University Hospital Basel, Basel, Switzerland

**Keywords:** blood pressure, hypertension, hypertensive disorders of pregnancy, preeclampsia, pregnancy

## Abstract

Pregnancy represents a unique physiological period characterized by dynamic cardiovascular adaptations and changes in blood pressure (BP) throughout gestation and the postpartum period. Deviations from these expected patterns may represent a risk to maternal health and be associated with the development of hypertensive disorders of pregnancy (HDP). Personalized and continuous monitoring, including BP measurements and the assessment of longitudinal and circadian BP patterns, can contribute to the earlier identification of women at risk. Accurate BP assessment remains central to the diagnosis and management of HDP. In this context, out-of-office BP assessment, particularly 24-hour ambulatory blood pressure monitoring, may provide additional information and identify abnormalities that remain undetected by conventional office measurements. HDP are among the most frequent complications of pregnancy and are associated with increased long-term cardiovascular risk. Their management extends beyond delivery, as postpartum hypertension remains underrecognized, and adequate follow-up and timely, targeted antihypertensive treatment are essential. Emerging strategies, including home BP monitoring, telemedicine-based programs, and multidisciplinary postpartum hypertension clinics, may improve continuity of care. This review examines physiological and pathological BP changes across pregnancy and the postpartum period, with particular emphasis on longitudinal BP trajectories and circadian patterns, beyond absolute BP values alone, as potential early indicators of HDP, while integrating current evidence and guideline recommendations on their diagnosis, monitoring, and management.

## Introduction

HT is the most prevalent cardiovascular (CV) disease worldwide and a major cause of global mortality and morbidity; hypertensive heart disease alone was responsible for an estimated 1.49 million deaths in 2023 (1). The period around pregnancy represents a window of opportunity in a women's health trajectory to prevent, diagnose, and treat HT as well as other CV risk factors. Indeed, it is now recognized that hypertensive disorders of pregnancy are major risk factors for future CV complications.

Hypertensive disorders of pregnancy (HDP) include chronic HT, gestational hypertension (GH), preeclampsia/eclampsia, and preeclampsia (PE) superimposed on chronic HT, with HELLP (Hemolysis, Elevated Liver enzymes, and Low Platelet count) syndrome representing one of the most severe clinical manifestations of PE. HDP are responsible for significant morbidity and mortality, both for the mother and her offspring. Importantly, HELLP syndrome may develop in the absence of the classic features of PE, as HT and proteinuria are absent in approximately 15% of cases (2). In the next decades, the incidence of HDP is expected to increase, driven by rising maternal age and the growing prevalence of cardiometabolic risk factors such as obesity and metabolic syndrome in the worldwide population (3). Beyond pregnancy, these conditions are strongly associated with an increased risk of long-term CV disease in affected women.

In this review, we aim to describe epidemiology, definitions, and diagnostic methods and criteria for HDP, as well as the main management strategies during pregnancy and after delivery.

## Epidemiology

Worldwide, HDP is estimated to occur in around 10% of pregnancies, and global PE incidence was estimated at 4.6% (4). These numbers vary by region, and socioeconomical status has also a negative impact on HDP prevalence and mortality (5). Despite a trend towards lower mortality, it is estimated that 16% of maternal deaths worldwide are still caused by HDP (6). The prevalence of HDP is the highest in Africa with 334 cases per 100,000 women of childbearing age, followed by South-East Asia and the Eastern Mediterranean region (7). A French cohort study found that 7.4% of pregnancies were complicated by HDP, with PE occurring in 2% and gestational HTN in 4.2% of pregnancies (8). A study found that globally, there has been an increase of 15% in the absolute number of HDP cases from 1990 to 2021, but a trend towards gradual decrease has been observed in the last years in regions with higher socioeconomical index (5). HDP and/or fetal growth restriction account for 48.4% of iatrogenic (medically indicated) preterm births (<34weeks), in England and Wales (9).

Major risk factors for HDP include: advanced maternal age (>35 years), nulliparity, obesity, multiple pregnancy, assisted reproductive technologies, diabetes mellitus (pre-existing or gestational), chronic HT, chronic kidney disease, autoimmune diseases, antiphospholipid syndrome, and a previous history of HDP, particularly PE (10–12). In a large meta-analysis including over 25 million pregnancies, antiphospholipid syndrome, chronic HT, and previous history of PE were associated with the highest risk of developing PE (13).

Postpartum HT is also common although probably underdiagnosed. It affects up to 8%–12% of women without antepartum HT and up to 50% of women with a history of PE within 6–12 weeks post-delivery (14–16). Most cases of *de novo* postpartum HT occur during the first week after birth, coinciding with the physiological postpartum blood pressure peak, and blood pressure returns to normal by 6 weeks in most women (13). However, reported incidence varies substantially according to the diagnostic definition, timing of blood pressure assessment, duration of follow-up, and study population. Furthermore, a systematic review and meta-analysis found that 28.4% of women with previous HDP were diagnosed with HT within 2 years postpartum, compared with 9.1% after normotensive pregnancies, indicating that the CV consequences of HDP may extend well beyond the conventional 6-week postpartum period (17).

Large prospective cohort studies have demonstrated that BP trajectories in early pregnancy are closely linked to long-term maternal CV risk. In a population of more than 170,000 women without preexisting HT, approximately 5% developed new-onset HT during extended follow-up (18). Women whose blood pressure remained stable or relatively elevated during early pregnancy—rather than exhibiting the expected physiological decline—were at increased risk of both HDP and subsequent chronic HT. This risk was further amplified in the presence of adverse pregnancy outcomes, including PE, preterm birth, or abnormal fetal growth (18). In particular, systolic BP patterns during the first 20 weeks of gestation showed a graded association with the future development of HT, spanning from the early postpartum period to more than a decade after delivery (18). These findings highlight that blood pressure assessment during pregnancy is not only relevant for immediate clinical management but also provides valuable prognostic information, allowing early identification of women at increased long-term CV risk and supporting the implementation of targeted preventive strategies.

## From physiological cardiovascular adaptation to pathological blood pressure trajectories

Pregnancy constitutes a unique physiological state characterized by profound and coordinated adaptations across multiple organ systems. From conception through delivery and into the postpartum period, maternal physiology undergoes complex cardiovascular, hormonal, and immunological changes driven by the evolving metabolic and hemodynamic requirements of the mother and fetus (19, 20). Among these, cardiovascular adaptations are particularly relevant to the regulation of maternal blood pressure.

Among these, CV adaptations are particularly significant. Pregnancy is characterized by increases in heart rate, stroke volume, and cardiac output, alongside a reduction in systemic vascular resistance (SVR) (20, 21). Structural and functional cardiac changes are also observed, including increased ventricular wall mass, enhanced myocardial contractility, and improved cardiac compliance. In uncomplicated pregnancies, the expansion of blood volume combined with decreased systemic vascular resistance establishes a high cardiac output, low-resistance circulatory state that ensures adequate uteroplacental perfusion (22–24). These hemodynamic changes begin early in gestation. During the first trimester, vasodilatory mediators such as nitric oxide, prostaglandins, and progesterone induce systemic vasodilation, leading to a reduction in systemic vascular resistance (SVR). Consequently, maternal blood pressure typically declines in early gestation. The decrease in SVR is compensated by a sustained rise in cardiac output, which rises by approximately 20%–40% over the course of pregnancy (19–21, 23, 24).

The increase in cardiac output facilitates redistribution of blood flow toward key organs, including the uterus, placenta, kidneys, and peripheral tissues. Enhanced uterine perfusion is essential for optimal placental development and function, thereby supporting adequate fetal vascularization, oxygenation, and growth (21). In early pregnancy, the rise in cardiac output is primarily driven by increased stroke volume, whereas in the third trimester, a progressive increase in heart rate becomes the predominant mechanism sustaining elevated cardiac output. This adaptation is necessary to meet the substantial rise in uterine blood flow, up to tenfold (19).

Renal hemodynamics are also profoundly altered during pregnancy. The increase in cardiac output is accompanied by augmented renal perfusion, resulting in a rise in glomerular filtration rate (GFR) of approximately 50% and an increase in renal plasma flow of up to 80% (19, 25). These hemodynamic adaptations result in lower serum concentrations of creatinine, urea, and uric acid (25). The renin–angiotensin–aldosterone system (RAAS) is markedly upregulated during normal pregnancy (24). Placental estrogen stimulates hepatic synthesis of angiotensinogen, leading to increased circulating levels of angiotensin II (24). Renin is produced not only by the kidneys but also by the ovaries and the decidua. Beginning at approximately 8 weeks of gestation, aldosterone concentrations rise progressively, reaching levels three- to sixfold higher than those observed in nonpregnant women by the third trimester. This promotes sodium and water retention, resulting in a net maternal extracellular fluid volume expansion of approximately 1.5 L (19, 25). Despite this pronounced activation of the RAAS, maternal blood pressure remains relatively low because vascular sensitivity to angiotensin II is reduced during normal pregnancy. In addition, progesterone acts as a physiological mineralocorticoid antagonist by decreasing aldosterone responsiveness at the receptor level, thereby attenuating sodium retention and contributing to the maintenance of normotension.

These coordinated hemodynamic adaptations determine the characteristic BP trajectory of uncomplicated pregnancy. Blood pressure typically declines during early gestation, reaching a nadir at approximately 20–24 weeks—a phenomenon often referred to as physiological hypotension (18, 26). Thereafter, BP gradually increases, returning toward prepregnancy levels by term. When these adaptive mechanisms are impaired, this physiological trajectory may become blunted or altered, potentially preceding the clinical development of HDP.

Maternal BP before conception and during early pregnancy is a major determinant of subsequent development of HDP. Women with higher baseline BP are indeed at higher risk of developing HDP, suggesting that pre-existing CV vulnerability influence susceptibility to these conditions (27).

Emerging evidence suggests that early hemodynamic alterations in pregnancies destined to develop HDP may not be adequately captured by conventional diagnostic critera, which rely primarily on absolute blood pressure thresholds. Longitudinal studies have demonstrated that women who subsequently develop GH or PE often remain normotensive during early pregnancy (26, 28). However, these pregnancies are characterized by a deviation from the expected physiological BP trajectory. While in healthy pregnancies BP progressively declines during the first half of gestation, reflecting physiological adaptation, this decrease appears blunted or absent in women who will subsequently develop hypertensive complications.

Regarding the physiological perspective, data from a large prospective cohort confirm that BP in uncomplicated pregnancies follows a characteristic and relatively narrow trajectory across gestation (29). This landmark international observational cohort study conducted between 2009 and 2016 across eight countries (Brazil, China, India, Italy, Kenya, Oman, the United Kingdom, and the United States) enrolled 4,607 healthy women at low risk of pregnancy complications (29). At approximately 12 weeks' gestation, median blood pressure values were around 111.5/69.1 mmHg. Diastolic blood pressure showed a slight decline, reaching a nadir of approximately 111/68.5 mmHg at around 20 weeks. Thereafter, both systolic and diastolic pressures progressively increased, reaching a median maximum of approximately 119.6/76.3 mmHg at 40 weeks' gestation, corresponding to an overall increase of about 8 mmHg systolic and 7 mmHg diastolic from early pregnancy to term (29).

Hermida et al. showed that women who later developed HDP already displayed subtle alterations in BP patterns early in pregnancy, despite having BP values within the normotensive range. During the first trimester, women who later developed HDP had a mean circadian BP of 115.3/67.2 mmHg, remaining well within the normal range but consistently higher than that of women with uncomplicated pregnancies. Hourly BP values also remained below 127/76 mmHg throughout the day. Despite these apparently “normal” BP levels, they failed to exhibit the physiological mid-gestational decline in BP typically observed in uncomplicated pregnancies (Figure 1) (28) @ (18, 24, 26–32, 33). This absence of physiological decline may represent an early marker of impaired CV adaptation, highlighting that altered BP trajectories, rather than absolute BP thresholds alone, may identify women at risk of HDP (24, 26, 29–32).

**Figure 1:**
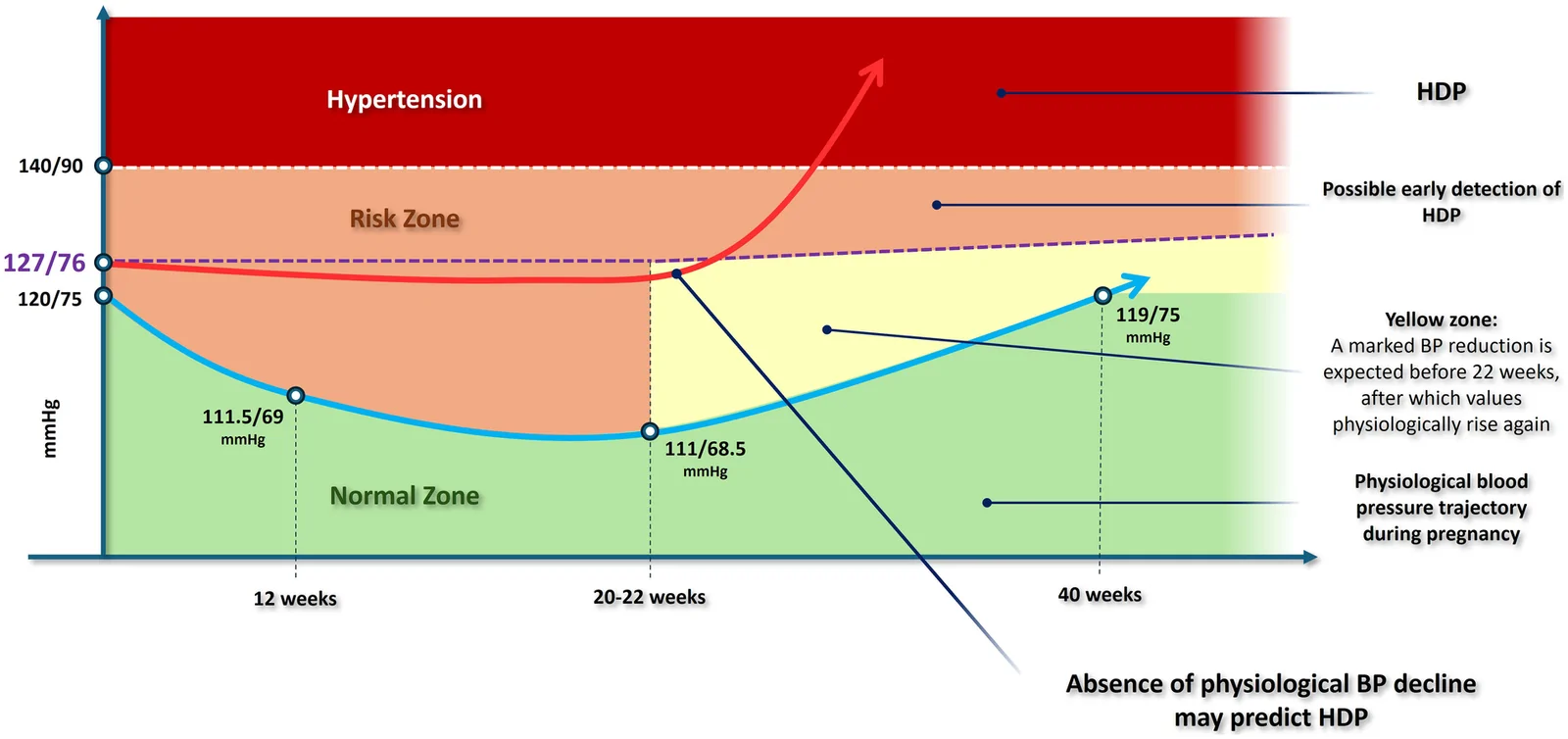
Longitudinal BP trends in pregnancy and early identification of women at risk of HDP (18, 24, 26–32, 33). HDP, hypertensive disorders in pregnancy; BP, blood pressure.

Indeed, this early alteration evolves into a distinct BP trajectory throughout pregnancy. Although BP generally remains within the normotensive range before the onset of overt HT, values are consistently higher than in uncomplicated pregnancies, with differences of approximately 13/7 mmHg (SBP/DBP) during the second trimester, increasing up to 16/9 mmHg during the third trimester, indicating a progressive divergence in maternal hemodynamic adaptation that precedes the clinical onset of HDP (26, 28).

The trajectory of BP alteration also differs according to HDP subtype, with PE characterized by a steeper rise in BP during the second half of pregnancy compared with GH (28, 32). Beyond absolute BP levels, alterations in circadian BP patterns may provide additional insights into disease progession. An early increase in circadian BP amplitude, reflecting greater day-night BP variability largely driven by nocturnal decline, has been observed before the onset of overt HT. This increase is followed by a reduction in circadian variability from the second to the third trimester, primarily due to a blunted nocturnal BP decline (28). This progressive alterations in circadian regulation may offer additional predictive value for identifying women at increased risk of more severe HDP phenotypes or manifestations, including the development of proteinuria.

Collectively, these findings support the concept that CV maladaptation precedes the onset of overt HT and suggest that integrating of circadian BP patterns with longitudinal BP trajectories, rather than relying on absolute BP values alone, may improve the early identification of women at risk and enhance the prediction of HDP (26, 28, 34, 35).

Among these circadian abnormalities, a blunted or absent nocturnal BP decline is commonly observed in women with PE and appears to be associated with neurohormonal dysregulation, including altered circadian activity of the RAAS and increased sympathetic activity (36, 37). These disturbances may contribute to the loss of normal circadian BP modulation observed before and during the development of PE. Consistent with this concept, nocturnal HT has emerged as a strong predictor of PE during the second half of pregnancy (≥ 20 weeks) (31, 32). In a large cohort study, both systolic and diastolic nocturnal HT remained independently associated with PE risk after adjustment for daytime BP and maternal characteristics (38), and women with combined systolic and diastolic nocturnal HT exhibited the highest risk. At mid-pregnancy screening (20–27 weeks), nocturnal HT was associated with a markedly increased risk of PE/eclampsia (OR: 5.32, 95% CI: 1.48–19.10) (38). Notably, when both daytime and nocturnal HT were incorporated into predictive models, only nocturnal HT remained statistically significant. These findings highlight the potential value of 24-hour ambulatory blood pressure monitoring (ABPM) with particular attention to nocturnal BP patterns for risk stratification in high-risk pregnancies, especially after 20 weeks of gestation.

Several mechanisms have been proposed to explain the attenuated nocturnal BP decline observed in PE. The loss of the expected nighttime reduction in vasoactive mediators, particularly in the presence of increased vascular reactivity, may contribute to elevated nocturnal blood pressure through impaired regulation of vascular tone and renal hemodynamics (36, 37). Another proposed mechanism involves altered sodium handling. In conditions characterized by salt sensitivity or subtle renal dysfunction, higher nocturnal BP may be required to facilitate sodium excretion or pressure natriuresis. In PE, despite the presence of edema, relative intravascular volume depletion coexists with sodium retention and systemic vasoconstriction. Under these conditions, the attenuation of nocturnal BP decline has been hypothesized to represent a compensatory response, that promotes sodium excretion while maintaining adequate placental perfusion (37, 39). Although early pregnancy values may remain within the normal range, subtle differences in absolute levels, circadian patterns, and temporal evolution can help identify women at increased risk. BP trajectories during pregnancy and key features associated with increased risk of HDP are summarized in Table 1 (19–39).

**Table 1:** Circadian blood pressure patterns by ABPM in physiological pregnancy and HDP: potential early markers of increased risk (19–39).

Phase	Expected in physiological pregnancy	Possible alterations suggesting increased risk of HDP
Early pregnancy (≤12 weeks)	Initiation of physiological BP decline Preserved or enhanced nocturnal dipping Gradual increase in circadian variability and amplitude	BP remains within normal range Early attenuation of nocturnal dipping (non-dipping or reversed pattern) Altered circadian amplitude (early increase followed by premature blunting)
Mid-pregnancy (20–24 weeks)	BP decline reaching nadir Preserved nocturnal dipping Stable circadian rhythm	Relatively higher BP levels, frequently in diastolic blood pressure (+13/7 mmHg) despite normotensive range Reduced or absent nocturnal dipping Early disruption of circadian variability
Second half of pregnancy (from 20 to 24 weeks)	Gradual BP increase from nadir within normal values Maintained circadian pattern	Higher BP levels (up to +16/9 mmHg) despite remaining in the normal range Blunted or reversed nocturnal decline Reduced circadian variability Steeper BP rise, particularly in preeclampsia

Given the physiological decline in BP during early pregnancy, the International Society for the Study of Hypertension in Pregnancy (ISSHP) proposes a 24-hour ABPM threshold of 127/76 mmHg at 18–22 weeks' gestation as a potential marker of increased risk for HDP. Reference ABPM values before 22 weeks are ≤126/76 mmHg for the 24-hour average, ≤132/79 for daytime, and ≤114/66 mmHg nighttime BP. Values above these thresholds may indicate the absence of the expected physiological BP decline during early pregnancy (Figure 2) (28, 30, 33) @ (28, 30, 31, 33, 40). By contrast, the European Society of Hypertension (ESH) and European Society of Cardiology (ESC) define HT in pregnancy as clinic BP ≥140/90 mmhg or home BP ≥135/85 mmHg and recommend to maintain a target diastolic BP of approximately 85 mmHg and systolic BP between 110 and 140 mmHg (30, 31, 40).

**Figure 2:**
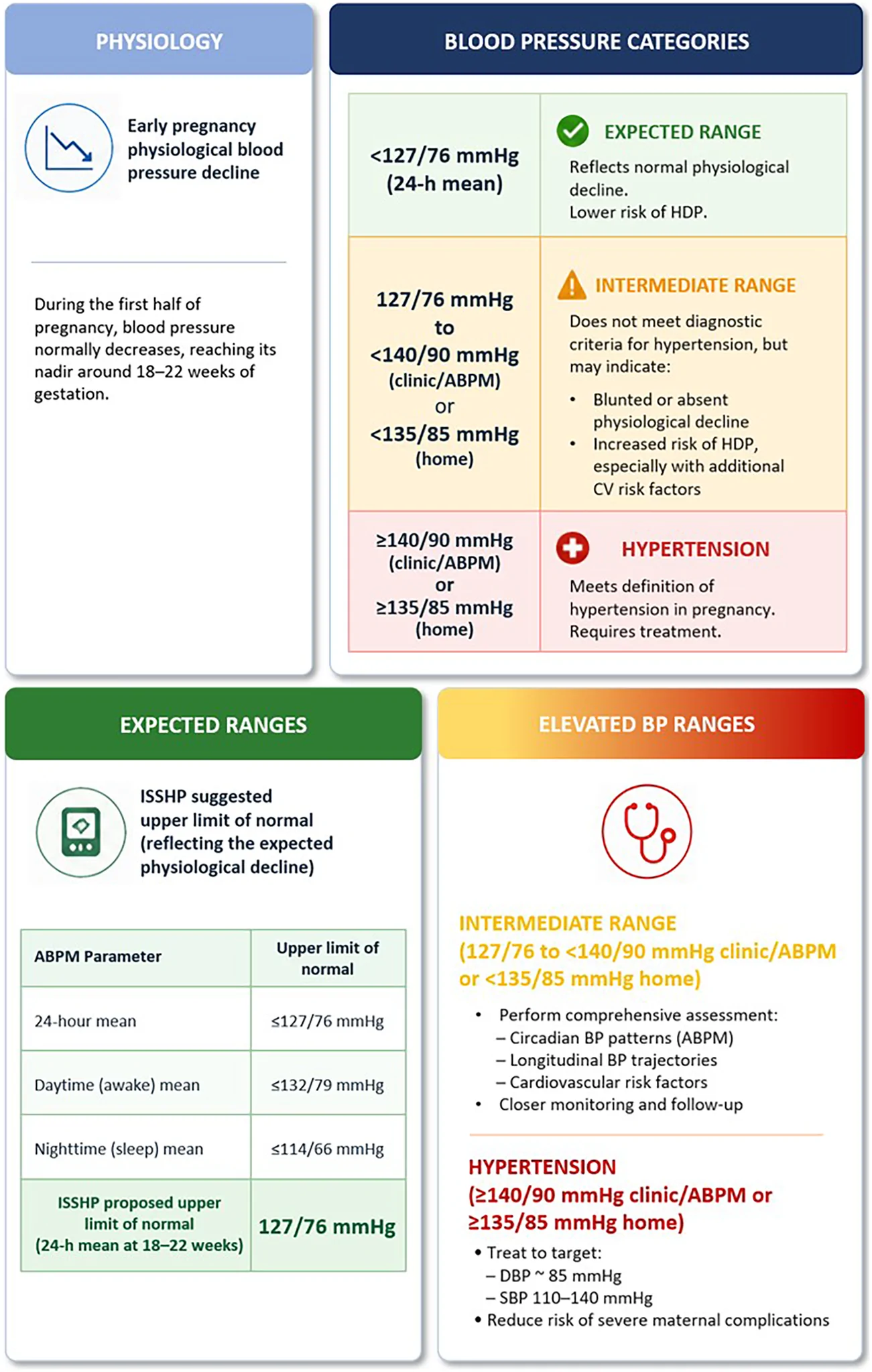
Blood pressure trajectories and thresholds in early pregnancy, from physiology to risk stratification and clinical management (28, 30, 31, 33, 40). ISSHP, international society for the study of hypertension in pregnancy (ISSHP); ABPM, ambulatory blood pressure monitoring.

Together, these recommendations highlight the existence of an intermediate BP range that falls below the conventional diagnostic threshold for HT but may nonetheless be considered as pathological and identify women at risk of developing HDP. However, particularly when accompanied by a blunted or absent physiological decline in BP during early gestation, they may reflect early CV maladaptation and identify women at increased risk, as consistently reported in both healthy and HDP pregnancy cohorts. Recognition of this “intermediate-risk” BP phenotype may provide an opportunity for closer surveillance and timely preventive interventions before overt HT or complications develops (26, 30, 31, 33, 37).

### Assessing blood pressure in pregnancy: diagnosis and monitoring approaches

Accurate BP assessment during pregnancy is essential to reduce adverse maternal and fetal outcomes, especially in women at risk of HDP. Although office BP measurement remains the standard and most widely used method (26, 28, 30, 31, 33, 40–41), out-of-office measurements-including 24h-ABPM and home blood pressure monitoring (HBPM)—provides a more accurate reflection of a patient's usual BP profile under real-life conditions than office measurements alone.

#### 24h-ambulatory blood pressure measurement (24h ABPM)

Day- and night-time ABPM provides the most comprehensive assessment by capturing both 24-hour BP levels and circadian patterns. This approach is particularly relevant during pregnancy, a period characterized by substantial physiological and temporal BP variations. Compared to office measurements, 24h ABPM derived BP may differ by up to +12/6 mmHg (SBP/DBP) (30, 31), suggesting the need for cautious interpretation of that office BP values in pregnant women. The European Society of Hypertension (ESH) 2024 position statement emphasises that 24h ABPM offers superior prognostic value compared with office and HBPM for predicting pregnancy outcomes, and that validated ABPM devices provide greater measurement accuracy (42). 24h ABPM also improves the detection of white-coat and masked HT, thereby reducing the risk of both unnecessary antihypertensive treatment and missed diagnoses. Furthermore, it may be particularly useful in the management of high-risk pregnancies, including those complicated by hypertensive disorders or diabetic nephropathy. However, despite these advantages, practical constraints limit its routine implementation. Consequently, HBPM and office BP measurements remain complementary tools in clinical practice, particularly when 24h ABPM is not feasible or readily available (40–42).

#### Home blood pressure monitoring (HBPM)

HBPM is increasingly used in the management of chronic HT and GH because of its accessibility and potential for longitudinal monitoring. However, current evidence has not demonstrated clear improvements in clinical outcomes compared with conventional clinic-based BP assessment alone. The recent BUMP-1 and BUMP-2 trials showed that self-monitoring neither improved the timing of HT diagnosis nor BP control (43, 44). Nevertheless, elevated home BP readings frequently preceded clinical diagnosis, suggesting that HBPM may provide an opportunity for earlier detection of BP abnormalities (43, 44). These findings suggest that self-monitoring alone is unlikely to improve outcomes but may offer clinical value when integrated into structured care pathways with predefined thresholds, timely review, and appropriate therapeutic interventions (45).

Beyond measurement strategies, improving outcomes also requires coordinated models of care. Innovative care pathways, such as postpartum transition clinics, dedicated HT follow-up services, and structured HBPM programs, have been developed to address the fragmentation that often affects the management of women with HDP (26). In parallel, telemedicine and emerging technologies, including cuffless monitoring devices, are increasingly being explored to facilitate BP assessment and monitoring, particularly during the postpartum period (46).

## Hypertensive disorders of pregnancy: from diagnosis to BP targets

A persistent area of controversy in HDP concerns the optimal BP threshold for initiating antihypertensive therapy. The therapeutic goal is not merely to reduce maternal BP but to achieve an appropriate balance between preventing maternal complications-such as severe HT, stroke, placental abruption- and preserving adequate uteroplacental perfusion to support foetal growth. This debate is rooted in the pathobiology of PE itself, which is widely recognised as a disorder of abnormal placentation and impaired placental vascular development. In this context, the rise in maternal systemic BP has historically been considered as a potential compensatory response aimed at maintaining placental and fetal perfusion (47). Consequently, treatment thresholds in pregnancy have traditionally been more conservative than those applied outside pregnancy, reflecting concerns that excessive BP reduction could compromise uteroplacental circulation. However, despite accumulating evidence supporting tighter control, variability remains across guidelines.

Two landmark trials have shaped this debate. In the CHIPS (48) trial, tighter control targeting a diastolic BP of 85 mmHg did not increase adverse perinatal outcomes but did substantially reduce the incidence of severe maternal HT compared with less-tight control. Subsequently, the CHAP (49) trial demonstrated that, among women with chronic HT, active treatment targeting BP below 140/90 mmHg reduced a composite of adverse maternal and perinatal events without increasing the risk of fetal growth restriction. Altogether, these findings shifted clinical practice away from the previous strategy of withholding treatment until server HT developed, particularly in chronic HT. However, the applicability of CHAP findings other HDP phenotypes, including GH and PE, remains less clearly established, contributing to persistent differences between guidelines regarding these conditions management.

Currently, major societies are aligned on diagnosis thresholds, although differences remain regarding treatment initiation and therapeutic targets. Indeed, most of them define HT in pregnancy as office BP ≥140/90 mmHg, and severe HT as ≥160/110 mmHg.

European recommendations have historically been heterogeneous, but more recent updates indicate a progressive alignment toward tighter blood pressure control. The 2024 position statement from the ESH reflects a shift toward earlier intervention, supporting the initiation of antihypertensive therapy from ≥140/90 mmHg in both chronic HT and GH, with a treatment target goal <140/90 mmHg but caution to avoid excessive diastolic reduction that could impair uteroplacental perfusion (42). This approach is also consistent with the latest 2025 guidance from the European Society of Cardiology, which defines HT in pregnancy as BP ≥140/90 mmHg and severe HT as ≥160/110 mmHg, based on repeated measurements. Management is guided by BP levels, gestational age, and maternal–fetal risk factors. Therefore, antihypertensive treatment is recommended for BP values between 140 and 159/90–99 mmHg with a target BP <140/90 mmHg, whereas severe HT warrants hospital admission and urgent management. Overall, contemporary European guidance supports earlier treatment initiation and consistent BP control, in line with emerging evidence from recent trials (41).

The main divergence concerns the timing of maintenance therapy for non-severe disease and the optimal BP targets once therapy has been started. NICE (50), ISSHP (30, 31), and SOGC (51) now recommend initiating earlier antihypertensive treatment for persistent HT at BP thresholds of 140/90 mmHg. In contrast, ACOG/SMFM (52) have adopted this threshold for the management of chronic HT following the CHAP trial but have not formally extended the same approach to all cases of GH or PE without severe features. SOMANZ recommends that women with gestational or chronic hypertension should have their BP controlled to a target of ≤135/85 mmHg (53) (Table 2) (30, 40–42, 49–53).

**Table 2:** Current international guidelines for diagnosis and BP targets in hypertensive disorders of pregnancy (30, 40–42, 50–53).

ESC-European Society of Cardiology/ESH-European Society of Hypertension
Diagnosis: hypertension in pregnancy defined as ≥140/90 mmHg; severe hypertension ≥160/110 mmHg, based on repeated measurements. ESC 2025 update: for BP 140–159/90–99 mmHg, antihypertensive treatment is recommended with a target <140/90 mmHg; severe hypertension (≥160/110 mmHg) requires hospital admission and urgent management. ESH 2024 update: initiation of treatment from ≥140/90 mmHg in both chronic and mild gestational hypertension, with a treatment target <140/90 mmHg and caution to avoid excessive diastolic BP reduction (particularly <80 mmHg).

ACOG-American College of Obstetricians and Gynecologists/SMFM-Society for Maternal-Fetal Medicine
Chronic hypertension: after CHAP, ACOG recommends using 140/90 mmHg as the threshold for initiation or titration of therapy. Gestational hypertension/preeclampsia: ACOG still centres urgent treatment on severe BP ranges, i.e., ≥160/110 mmHg, and has not formally extended the CHAP threshold to all non-severe gestational hypertension.

NICE-National Institute for Health and Care Excellence
Treatment initiation: offer antihypertensive therapy if BP remains above 140/90 mmHg. Target on treatment: ≤135/85 mmHg.

ISSHP-International Society for the Study of Hypertension in Pregnancy
Treat hypertension to a target diastolic BP of 85 mmHg; treatment of non-severe hypertension is recommended from around 140/90 mmHg, prior to development of severe values. Home BP threshold: 135/85 mmHg.

SOGC-Society of Obstetricians and Gynaecologists of Canada
Treatment initiation: 140/90 mmHg regardless of HDP subtype. Target: DBP 85 mmHg; a similar target may be considered in preeclampsia. Severe hypertension: urgent treatment at ≥160/110 mmHg.

SOMANZ-Society of Obstetric Medicine of Australia and New Zealand
Diagnosis: ≥140/90 mmHg. Target for chronic or gestational hypertension: SBP ≤135 mmHg and DBP ≤85 mmHg. Acute severe hypertension: treat to <160/110 mmHg.

BP, blood pressure; DBP, diastolic blood pressure; CHAP, chronic hypertension and pregnancy project trial (49).

In clinical practice, early BP alterations in women who subsequently develop HDP often involve a preferential increase in diastolic values, which may precede detectable rises in systolic BP, particularly during the early and mid-gestational periods. This pattern likely reflects an early increase in peripheral vascular resistance and subtle impairment of vascular adaptation, occurring before BP values exceed conventional diagnostic range (28, 54).

Looking ahead, a more personalized approach to care may represent the most effective strategy for managing HDP. In a recent study, patients with chronic HT, and pre-existing diabetes before 20 weeks' gestation, who achieved an average goal BP of <130/80 mmHg experienced improved perinatal outcomes compared to those with higher BP levels (55). Lower BP was associated with reduced rates of severe PE, indicated preterm birth, and neonatal intensive care unit admission, without increasing cesarean delivery, fetal or neonatal death, or fetal growth restriction, which appears to be less frequent in this group (55). These findings suggest that, in carefully selected high-risk populations, lower BP levels targets may be both safe and beneficial, supporting the interest of a more individualized approach in antihypertensive treatment. Although maternal and perinatal outcomes are generally favourable in GH, up to one-quarter of women—especially those diagnosed before 34 weeks' gestation-may develop PE, resulting in a substantially worse prognosis (30, 31, 40–42). This highlights the need for risk-stratified management that incorporates not only BP levels but also gestational age at presentation, underlying maternal risk factors, and the likelihood of disease progression.

### Prevention and pharmacological management of HDP

Low-dose aspirin (75–150 mg daily) remains the only established pharmacological strategy for the prevention of PE. It is recommended for women at increased risk, including those with chronic HT, a history of PE, or conditions such as pregestational diabetes, chronic kidney disease, autoimmune disorders (e.g., systemic lupus erythematosus or antiphospholipid syndrome), multifetal pregnancy, assisted reproduction, advanced maternal age, or a family history of PE (26, 47). Once PE develops, prompt treatment of HT is essential to reduce maternal morbidity and mortality and to minimize fetal complications, particularly when the condition arises during the antenatal period.

First-line antihypertensive agents considered safe during pregnancy include labetalol, calcium channel blockers (such as nifedipine or amlodipine), and methyldopa (26, 30, 31, 40, 47). Thiazide diuretics, such as hydrochlorothiazide, may also be used in selected cases, with careful monitoring of amniotic fluid volume (41). Other agents, including verapamil, can be considered as third-line therapy (26) (Table 3) (26, 30, 31, 40, 41, 47, 56–59). There is emerging interest in the use of mineralocorticoid receptor antagonists (MRA) in selected clinical scenarios, such as severe hyperaldosteronism during pregnancy (26, 41, 56, 57). Amiloride, a potassium-sparing diuretic acting as a direct epithelial sodium channel inhibitor, is considered relatively safe and already used in conditions such as Bartter and Gitelman syndromes (58). Eplerenone and spironolactone remain less well studied. In general, available data suggest that MRA use may be considered from the second trimester, particularly in pregnancies with a female fetus, as exposure during early gestation may interfere with male genital development (57, 59).

**Table 3:** Pharmacotherapy for pregnancy and postpartum hypertension (26, 30, 31, 40, 41, 47, 56–59).

Treatment line	During pregnancy	Postpartum during lactation
First-line treatment	**Labetalol** **Nifedipine/Amlodipine** **Methyldopa**	**Nifedipine/Amlodipine** **Enalapril** (preferred if high cardiovascular risk or proteinuria) **Labetalol**
Second-line treatment	**Hydrochlorothiazide** (with monitoring of amniotic volume) **Hydralazine** **Clonidine**	**Hydrochlorothiazide** **Eplerenone**/**Spironolactone** (both with monitoring of milk volume production)
Third-line, in very specific conditions and with precise monitoring	**Verapamil** **Amiloride** (preferred, from 2nd to 3rd trimester) **Eplerenone**/**Spironolactone** (only in selected case, from 2nd to 3rd, safer if female)	**Captopril** **Candesartan** **Torasemide**/**Furosemide** (with monitoring of milk volume production)

Drug names are provided in bold to improve readability.

## Postpartum blood pressure management and maternal cardiovascular risk

HDP are now recognized as major risk factors for subsequent development of chronic HT and long-term CV disease (11, 60). Beyond BP control, emerging evidence suggests that early postpartum antihypertensive strategies may have favorable effects on maternal cardiovascular health.

In a nationwide Danish cohort, the excess risk of chronic HT emerges early after an HDP-affected pregnancy, with incidence rates 12- to 25-fold higher during the first postpartum year, 3- to 10-fold higher between 1 and 10 years postpartum, and remaining approximately twofold higher even more than 20 years after delivery (61). These findings are consistent with evidence from a systematic review and meta-analysis including more than 600,000 women, which reported that a history of HDP was associated with a 3.6-fold higher risk of developing chronic HT compared with normotensive pregnancies. The association was strongest after GH (6.2-fold increase), whereas women with previous PE had a 3.2-fold higher risk (62).

PE is associated with a four-fold increased risk of heart failure and a two-fold increased risk of death from coronary heart or other CV disease (63). Although all subtypes of HDP increase the risk of CV disease, particularly heart failure and stroke, recent evidence suggests that superimposed PE confers the greatest CV burden (64).

A long-term cohort study reported that women with a history of HDP had an increased risk of premature all-cause mortality (adjusted HR 1.31, 95% CI 1.18–1.46), with the strongest association observed for cardiovascular mortality (HR: 2.26, 95% CI: 1.67–3.07) (65). This risk was greater among women with both HDP and chronic HT, than among those with chronic HT alone. Importantly, the incidence of stroke, heart failure and coronary heart disease was found to be higher during the first postpartum decade, a period during which women are often no longer considered at increase CV risk, particularly when their obstetric history is not routinely incorporated into CV risk assessment (63).

The mechanisms linking HDP to future CV disease are likely multifactorial. HDP and CV disease share several established CV risk factors. Pregnancy has been proposed as a physiological "stress test", that may unmask an underlying predisposition to future HT and CVD. However, accumulating evidence suggests that HDP is not merely a marker of pre-existing CV risk but may itself contribute to subsequent CV disease, independently of traditional cardiometabolic risk modifiers. Consistent with this hypothesis, the excess risk of future chronic HT remains elevated even after adjustment for these risk factors (62, 66). Although the underlying mechanisms are not yet fully understood, persistent endothelial dysfunction, angiogenic imbalance, chronic inflammation, complement activation, epigenetic modifications, and vascular remodeling have all been proposed as potential contributors (60).

In the postpartum period, calcium channel blockers, labetalol and angiotensin-converting enzyme (ACE) inhibitors, such as enalapril, are considered as first-line therapy (26, 30, 31, 40, 41). These agents are compatible with, while ACE inhibitors may provide additional cardioprotective, nephroprotective, and antiproteinuric effects. Supporting these cardiovascular benefits, the PICk-UP trial showed that 6 months of enalapril treatment following preterm preeclampsia was associated with improved diastolic function and cardiac remodeling compared with placebo. Women receiving enalapril also showed a lower prevalence of persistent concentric remodeling or hypertrophy (26% vs. 57%), together with lower diastolic BP (67). More recently, ESC guidelines have suggested a potential role for candesartan in the immediate postpartum setting, although further evidence is required to support its routine use (41).

With regard to BP monitoring and the intensity of postpartum follow-up, emerging evidence supports a more structured and proactive approach to BP surveillance and treatment adjustment. In this context, telemedicine may facilitate frequent assessment and timely modification of antihypertensive therapy. The POP-HT randomized clinical trial evaluated a strategy combining daily home BP self-monitoring, remote transmission of BP measurements, and physician-guided antihypertensive titration in women following a hypertensive pregnancy. Compared with usual postpartum care, this intensified management approach resulted in better 24-hour ambulatory BP control at approximately 9 months postpartum and was associated with fewer hypertension-related readmissions within the first 14 days after discharge (7% vs. 27%). These findings support the value of integrating remote monitoring within a proactive and structured postpartum management pathway (68).

## Conclusions

HDP are increasingly prevalent, partly reflecting the rising maternal age at conception, and represent a major cause of maternal and neonatal morbidity, with important long-term CV implications for both mother and child. While diagnostic criteria for hypertension are well established, heterogeneity remains in the assessment and management of HDP, and recommendations from international societies are not fully aligned. In addition, therapeutic options continue to be limited.

Early identification of women at risk remains a major clinical challenge. Current evidence suggests that 24-hour ABPM provides the most comprehensive tool for assessment. Beyond average BP values, increasing attention should be paid to circadian patterns and longitudinal BP trajectories, which may identify impaired CV adaptation before overt HT develops and could facilitate early diagnosis and personalized management.

The question of the right threshold to initiate antihypertensive therapy remains an area of debate, requiring a careful balance between reducing maternal complications from severe HT and preserving adequate uteroplacental perfusion. Nevertheless, the landmark CHAP trial has shifted clinical practice toward earlier blood pressure control in women with chronic HT, demonstrating improved maternal and obstetric outcomes without increasing the risk of fetal growth restriction or adverse neonatal outcomes.

Maternal cardiometabolic risk factors during pregnancy are consistently linked to higher BP in offspring from early life, as well as to a more pronounced increase over time. Among these, HDP appear to play a central role.
